# Enhancement of doxorubicin efficacy by diosmetin through DNA damage accumulation and P-glycoprotein inhibition in breast cancer cells

**DOI:** 10.1038/s41598-025-16681-3

**Published:** 2025-08-21

**Authors:** Monika Michalczyk, Ewelina Humeniuk, Joanna Kubik, Grzegorz Adamczuk, Mariola Michalczuk, Barbara Madej-Czerwonka, Maciej Czerwonka, Agnieszka Korga-Plewko

**Affiliations:** 1 Independent Medical Biology Unit, Medical University of Lublin, 8b Jaczewskiego, Lublin, 20-090 Poland; 2 Human Anatomy Department, Faculty of Medicine, Medical University of Lublin, 4 Jaczewskiego, Lublin, 20-090 Poland; 3 First Department of Surgery, Jagiellonian University Medical College, 2 Jakubowskiego, Krakow, 30-688 Poland

**Keywords:** Diosmetin, Diosmin, Doxorubicin, DNA damage, *ABCB1* activity, Breast cancer, DNA damage and repair, Apoptosis

## Abstract

Doxorubicin (DOX) is widely used in treatment; however, its efficacy is often compromised by multidrug resistance (MDR), limiting therapeutic outcomes. This study investigates the anticancer potential of diosmetin (DT), a metabolite of diosmin commonly used in chronic venous insufficiency, and its ability to enhance the efficacy of DOX in the treatment of breast cancer. Several breast cancer cell lines were used to assess the effects of DT and DOX on cell viability. The MCF-7 cell line, which showed the most significant effect, was selected for further investigation. The effects of DT on apoptosis, DNA damage, and P-glycoprotein expression and activity were analyzed. DT significantly enhanced DOX-induced apoptosis and DNA damage, while downregulating ABCB1 expression and activity, a key factor in MDR development. These findings highlight the potential of DT as a valuable adjuvant in breast cancer therapy by enhancing the efficacy of DOX. Its ability to overcome multidrug resistance mechanisms underlines its potential for future clinical trials aimed at developing effective treatments.

**Supplementary Information:**

The online version contains supplementary material available at 10.1038/s41598-025-16681-3.

## Introduction

Breast cancer represents one of the most frequently diagnosed malignancies worldwide and is the primary cause of death among women^1^. Furthermore, Arnold et al. (2022) estimated an increase in newly diagnosed breast cancer incidence by more than 40% and a 50% increase in mortality by the year 2040^2^. Thus, breast cancer presents a significant healthcare and socioeconomic challenge.

Doxorubicin (DOX) is a commonly prescribed anthracycline chemotherapeutic agent in the treatment of metastatic breast cancer^3,4^. However, DOX has a clinical efficacy rate of 40% due to the development of resistance during treatment^5^. The primary factor responsible for multidrug resistance (MDR) in cancer is the overexpression of several ATP-binding cassette (ABC) transporters located on the cell membrane. These proteins are linked to an energy-consuming pump to remove natural cellular metabolites or harmful chemicals from the cells. Overexpression of ATP-binding cassette transporters, particularly P-glycoprotein (P-gp), is a major contributor to MDR in breast cancer^6,7^. P-gp-ABCB1 reduces intracellular drug concentrations and mitigates their toxicity by facilitating the removal of anticancer drugs, including DOX, from cancer cells^8^. Suppression of ABC transporters could potentially overcome MDR and reverse the diminished anticancer properties of standard chemotherapeutic agents^9–11^.

Naturally occurring dietary components, including flavonoids, have been found to effectively counteract MDR. Indeed, many flavonoids have been shown to suppress DOX resistance in tumor cells by blocking certain ABC transporters, enhancing the efficacy of DOX, preventing the emergence of MDR. In the group of flavonoids, diosmetin (DT) has been shown to have antitumor effects by controlling cell cycles, triggering apoptosis, and thus suppressing tumor growth in a variety of cancers^3–9^. DT is the flavone aglycone and major metabolite of diosmin, a widely used drug for conditions related to venous insufficiency^10,11^. When diosmin is administered, intestinal microflora enzymes hydrolyze it to DT and facilitate bloodstream absorption. Hence, DT constitutes the bioactive form of diosmin^12^. Furthermore, research indicates that DT itself, when administered directly, exhibits better bioavailability than diosmin. This is due to its smaller molecular size and higher lipophilicity, which allow for more efficient absorption without the need for metabolic conversion^13^. Chronic venous insufficiency is a common disease, especially in developing countries^19^. It has been shown that breast cancer and chronic venous insufficiency appear to be strongly associated. Oncological treatment may predispose to a more severe clinical course of venous disease when present in patients with breast cancer^14,15^.This situation undoubtedly influences the popularity of preparations containing diosmin. The described properties of these substances suggest that the use of preparations that improve blood flow may be important in the prevention or treatment of many types of tumours. Because of the wide range of possible biological activities of this flavonoid, this effect is not obvious. It has not yet been established how the drugs commonly used to treat chronic venous insufficiency, which is also common in breast cancer patients, affect the development of breast cancer and chemotherapy, including DOX treatment.

Therefore, the purpose of this study is to explore the impact of DT on breast cancer cells both as a monotherapy and in combination with DOX. The effects of DT on cell viability, apoptosis, and DNA damage were investigated. In addition, we examined its effect on ABC transporter expression and activity to better explain the observed interactions. The findings of this study are anticipated to provide a foundation for future research into the prospective application of DT as a viable co-chemotherapeutic agent in breast cancer therapy.

## Materials and methods

### Cell culture and treatment

Four breast cancer lines (MCF-7, MDA-MB231, MDA-MB460, T47-D) were used in this study (ATCC, USA). The MCF-7 cells were cultured in Eagle’s minimum essential medium (EMEM) (Corning, USA), MDA-MB231, and MDA-MB460 were cultured in Kaighn’s Modification of Ham’s F-12 Medium (F12K) (Corning, USA), and T47D and ZR7 cell lines were cultured in RPMI (Corning, USA), supplemented with 10% fetal bovine serum (Corning, USA), and incubated at 37 °C with 5% CO2 in an air atmosphere.

The cells were treated for 48 h with a range of DT concentrations(Sigma-Aldrich, USA) − 20, 40 and 80 µM and DOX (EBEWE Pharma, Austria) − 0.1, 0.5 and 1µM or a combination. In the control groups, cells were treated with DMSO (0.5%), which served as a vehicle for the treatment.

### Cytotoxicity analysis

An MTT assay was performed to determine cell viability. The test relies on the ability of live cells to transform the orange tetrazolium salt (3-(4,5-dimethylthiazol-2-yl)−2,5-diphenyltetrazolium bromide) (Sigma-Aldrich, USA) into purple formazan crystals, which are insoluble in water. Hence, the quantity of formazan generated corresponds to the quantity of living cells. For all the assays, the cells were seeded into 96-well plates at a concentration of 2 × 10^4^ cells per well. When 70–80% confluence was reached, the tested compounds were added. After a 48-hour incubation period, the prepared MTT solution (0.5 mg/mL in phosphate-buffered saline) was added to each well. Following a 4-hour incubation period, the MTT medium was discarded and the crystals formed were dissolved in DMSO. Control cultures were treated with DMSO (0.5%) as a vehicle. Quantification of the solution’s absorbance was conducted at 570 nm wavelength through the use of a PowerWave XS microplate spectrophotometer (BioTek Instruments, USA). Each assay was conducted three times and was evaluated in triplicate.

### Apoptosis detection

The evaluation of apoptosis was conducted employing the NucleoCounter NC-3000 (ChemoMetec, Denmark) and Annexin V Apoptosis Assay (ChemoMetec, Denmark). The cells were seeded into 6-well plates at a concentration of 4 × 10^5^ cells/well, and the tested compounds were added when 70–80% of confluence was achieved. Following 48 h of incubation with the examined substances, the cells were harvested using trypsin-EDTA solution (Corning, USA), and stained with Annexin V-FITC (fluorescein isothiocyanate), Hoechst 33,342, and propidium iodide by the manufacturer’s recommended protocol. The cell suspension (6 × 10^4^/30µl) was loaded into the NC-Slide and read in the NucleoCounter NC-3000. The gating strategy was established based on forward and side scatter properties to exclude debris, followed by quadrant analysis on FITC and PI fluorescence to identify distinct cell populations. Unstained and single-stained controls were used to set compensation and define the gates accurately. This approach allowed precise quantification of apoptosis across experimental groups. Each experiment was conducted in triplicate, with three independent repetitions, following the previously established methodology^16,17^.

### Comet assay

This approach is utilized to identify the genotoxic substances. MCF-7 cells were seeded as above (in 6-well plates at a concentration of 1 × 10^5^ cells/well) and treated with DOX (1 µM), and DT (80 µM) and their combination of both for 48 h. The cells were collected by trypsinization using a trypsin–EDTA solution (Corning, USA), and washed with PBS. Each sample’s final concentration of MCF-7 cells was adjusted to 1 × 10^5^. The comet assay was conducted under neutral conditions (pH 8.5), as described previously by Olive et al., 2006^18^. The cell suspensions (0.4 mL) were mixed with 1.2mL of 1% (w/v) low melting point agarose (LMA), and then distributed onto microscope slides coated with 1% (w/v) normal melting agarose, covered with a cover slip, and kept for 10 min at 4 °C to solidify. Then, the microscope slides were submerged in a covered dish containing N1 lysis solution (2% sarkosyl, 0.5 M Na_2_EDTA, 0.5 mg/ml proteinase K (pH 8.0) (ThermoFisher, USA) and kept at 37 °C overnight in the dark. After overnight lysis, the slides were two times submerged in room temperature N2 rinse buffer (90 mM Tris buffer, 90 mM boric acid, 2 mM Na2EDTA (pH 8.5)) for 30 min and placed in an electrophoresis tank horizontally side by side. Electrophoresis in solution N2 was run for 25 min at 0.6 V/cm. After electrophoresis, the slides were washed for 15 min in distilled water, and stained using Hoechst 33342 (5 µg/mL) for another 20 min and excess stained slides were rinsed with 400 ml of distilled water Comet images were captured using a Nikon Eclipse Ti inverted microscope with NIS-Elements Imaging Software (Nikon, Japan). One hundred individual cells were selected for calculations for each analysis. The slides were examined under a fluorescence microscope with a filter that matched the previously applied fluorescent dye. The images were analyzed using OpenComet (v1.3) a plugin for the image processing program ImageJ^19^ which gives % DNA in tail, tail length, and tail moment (TM), directly. The parameter TM is the product of tail length and % DNA in the tail. The software determines the fluorescence intensity by calculating many factors that describe the extent of DNA damage in each cell. The proportion of DNA in the comet’s “head” represents genetic material in the nucleus, while the fluorescent area in the “tail” indicates genetic material that has moved out of the cell nucleus during electrophoresis. Tail length, and Olive moment were used as evaluations of the DNA damage.

### Molecular docking simulations

To explore the interaction between DT and the P-gp receptor, molecular docking technology was used. Specifically, AutoDock 4.2 molecular docking software was applied for semi-flexible docking between DT and P-gp in this study. Semi-flexible docking means that the receptor is rigid, while the ligand structure may change within a certain range. The docking model was performed based on the 3D crystal structures of human P-glycoprotein and was downloaded from the Protein Data Bank (PDB) (PDB code: 6C0V). Ligands and proteins were pre-paired using a previously established approach^20^. The preparation of the selected protein involved removing water molecules and all co-crystallized entities from the protein structures. Additionally, polar hydrogen atoms were added to the enzyme structures, nonpolar hydrogen atoms were merged, and Kollman charges were assigned and distributed over the residues. All preparation procedures were performed using AutoDock Tools v.1.5.7^21^. The docking parameters are given in Table 1.

**Table 1:** Molecular Docking parameters. AutoDock 4.2 molecular Docking software was used for semi-flexible Docking between DT and P-gp. The tested protein was treated as rigid while keeping the ligand flexible. P-gp: P-glycoprotein; DT- diosmetin.

Size of girid box	100 × 100 × 100
Girid points spaced	0.375
Algorithm	Lamarckian genetic algorithm (LGA)
Genetic algorithm runs	30
Population size	300 inviduals
Maximum numer of Energy evaluations	250,000
Gegenerations	150,000
Mutation rate	0.02
Crossover rate	0.80

The conformations with the lowest binding energies were chosen for comparison with the docking results of the reference ligands. Positions with a root mean square deviation (RMSD value (≤ 2.0) confirm the relevant predictions for the compounds of interest^22^. We obtained 0,936 Å, which indicated that the parameters in the docking simulations were acceptable (Fig. 4c). Discovery Studio Visualizer v.21.1. was used for visualization and interpretation of the received data^23^. To validate the docking protocol and provide a comparative basis, known P-gp inhibitor verapamil was docked to the same P-gp structure (PDB ID: 6C0V) as reference compounds^24^. The docking results of DT were compared to these controls based on binding energy and estimated inhibition constants (Ki). These comparisons help contextualize DT’s predicted inhibitory potential.

### Hoechst accumulation assay

The cells were seeded into 6-well plates at a concentration of 4 × 10^5^ cells/well, and the tested compounds were added when 70–80% of confluence was achieved. To measure the activity of of P-gp in the tested MCF-7 cells Hoechst Accumulation Assay was performed using the Hoechst 33,342 (5 µg/mL) which is a fluorogenic dye. After 48 h of incubation with the tested substances, the cells were stained with Hoechst 33,342 (ThermoFisher, USA) Subsequently, the cells were subjected to incubation at 37 °C for 30 min. Following the incubation, the cells were washed three times with PBS and were then visualized using a fluorescence microscope on a Nikon Eclipse Ti inverted microscope with NIS-Elements Imaging software NIS-Elements BR, version 3.22 (Nikon, Japan, https://www.microscope.healthcare.nikon.com/products/software/nis-elements). For each sample, 3 random fields of view were selected using a 20× objective lens. In each field of view, 20 nuclei were analyzed, ensuring that the nuclei were clearly stained, non-overlapping, and fully visible within the analyzed area. Nuclei with atypical morphology (e.g., fragmented or unevenly stained) were excluded from the analysis. Regions of Interest (ROI) corresponding to the nuclei were automatically defined using the software’s thresholding and segmentation tools, optimized for the Hoechst 33,342 fluorescence channel. For each automatically defined ROI, the mean fluorescence intensity (Mean Intensity) was measured in the channel corresponding to Hoechst 33,342 emission. The procedure was repeated for 3 biological replicates, with each field of view analyzed independently. In total, the fluorescence intensity of 60 nuclei per sample (3 fields of view × 20 nuclei) was measured. The recorded mean fluorescence intensity values were saved for further statistical analysis.

### The quantitative real-time PCR analysis (qRT-PCR)

The cells were seeded into a 25-cm^3^ flask at a concentration of 4 × 10^4^ cells/mL. The tested compounds were added when 70–80% of confluence was achieved. After 48 h of incubation, 1 mL of TRIzol™ Reagent (Invitrogen, USA) was added to the culture dish to lyse the cells. Afterward, the lysate was centrifuged for 5 min at 12,000× g at 4 °C, and then the clear supernatant was treated in compliance with the producent’s protocol. The obtained RNA was quantified and normalized to equal concentrations prior to reverse transcription, which was performed using the NG dART RT-PCR kit (EURx, Poland) in accordance with the manufacturer’s instructions. The qPCR was performed using SG/ROX qPCR Master Mix reagents (2x) (EURx, Poland) based on the manufacturer’s instructions in a 7500 Fast Real-Time PCR System (ThermoFisher, USA). The reaction was conducted in triplicate. The procedure was repeated for 3 biological replicates. The relative expression of the tested genes was performed by qRT-PCR and the ΔΔCt method using *18SRNA* and *ACTB* as reference genes. A statistical analysis was determined with RQ values (relative quantification, RQ = 2^−∆ΔCt^). The primer sequences are summarized in Table 2.

**Table 2:** Primers used in the assessment of gene expression.

Target	Forward Sequence (5′→3′)	Reverse Sequence (3′→5′)
***ABCB1***	TCTTCACCTCCAGGCTCAGT	GCTCCTGACTATGCCAAAGC
***18SN5***	GCAGAATCCACGCCAGTACAAG	GCTTGTTGTCCAGACCATTGGC
***ACTB***	CACCATTGGCAATGAGCGGTTC	AGGTCTTTGCGGATGTCCACGT

### Analysis of P-glycoprotein expression by western blotting

The cells were seeded into a 25-cm^3^ flask at a concentration of 4 × 10^4^ cells/mL. The tested compounds were added when 70–80% of confluence was achieved. After 48 h, the cells were washed twice with cold PBS. Next, 1 ml of cold RIPA buffer (ThermoFisher, USA) was added to the cells and the protein was isolated according to the manufacturer’s instructions. the protein concentration in the supernatants was determined by the Bradford method. The extracted protein (20 µg) was loaded in a polyacrylamide gel (NuPAGE Bis-Tris Gels, Invitrogen, USA) and 50-min electrophoretic separation was performed under reducing conditions with XCellSureLock instrument (Invitrogen, USA) at a constant voltage of 200 V. After separation, the gel and the nitrocellulose membrane (Invitrogen, USA) were placed between two layers of the filter paper and put between two electrodes of the XCell II Blot Module electrotransfer apparatus (Invitrogen, USA). The transfer was conducted for 60 min at a constant voltage of 30 V. Blots were developed using Western Breeze Chromogenic Detection Kit (Invitrogen, USA). For simultaneous detection of different primary antibodies, membranes were carefully cut horizontally before antibody incubation. Nonspecific antibody-binding sites were blocked and the membrane was incubated in the solution of the primary antibody: P-Glycoprotein Polyclonal Antibody (PA5-87086, ThermoFisher, USA) or β-actin antibody (N-21) (Santa Cruz Biotechnology, USA). After washing, the membrane was incubated with the secondary antibody combined with alkaline phosphatase. The last step was to mark the sites of reaction with antigen by the reaction of alkaline phosphatase and chromogen. Band intensities were quantified by densitometric analysis with the Image Studio 6.0 Software (Li-Cor, USA) and normalized to β-actin as a loading control. Before analysis, the blot images were converted to grayscale. Values were expressed as the ratio of target protein to β-actin band intensity.

### Statistical analysis

The statistical analysis was performed using the STATISTICA 13 software (StatSoft, Poland). The normality of the variable distribution was verified using the Shapiro–Wilk test. The data were analyzed using Two-way analysis of variance (ANOVA) with DOX and DT80 treatments (presence/absence) as fixed factors. The model also included the interaction term between both treatments. Post-hoc multiple comparisons were conducted using Tukey’s honest significant difference test. The correlation between tail length and tail moment in the comet assay was assessed using Pearson’s correlation coefficient. Results were reported as mean ± standard deviation (SD) and statistical significance was defined at *p* < 0.05. All experiments were performed in three independent biological replicates, each including s technical replicates.

Synergy analysis of DOX and DT was performed using Combenefit (Cancer Research UK Cambridge Institute). The percent inhibition values were used to construct an effect matrix which was analysed using the Highest Single Agent (HSA) model. This model assumes that the effect of the combination should not exceed that of the more potent drug in the combination. Heat maps of drug-drug interactions and quantitative synergy indices were obtained to assess the nature and strength of the interaction of the compounds studied^25^.

## Results

### Cytotoxicity analysis

**Fig. 1:**
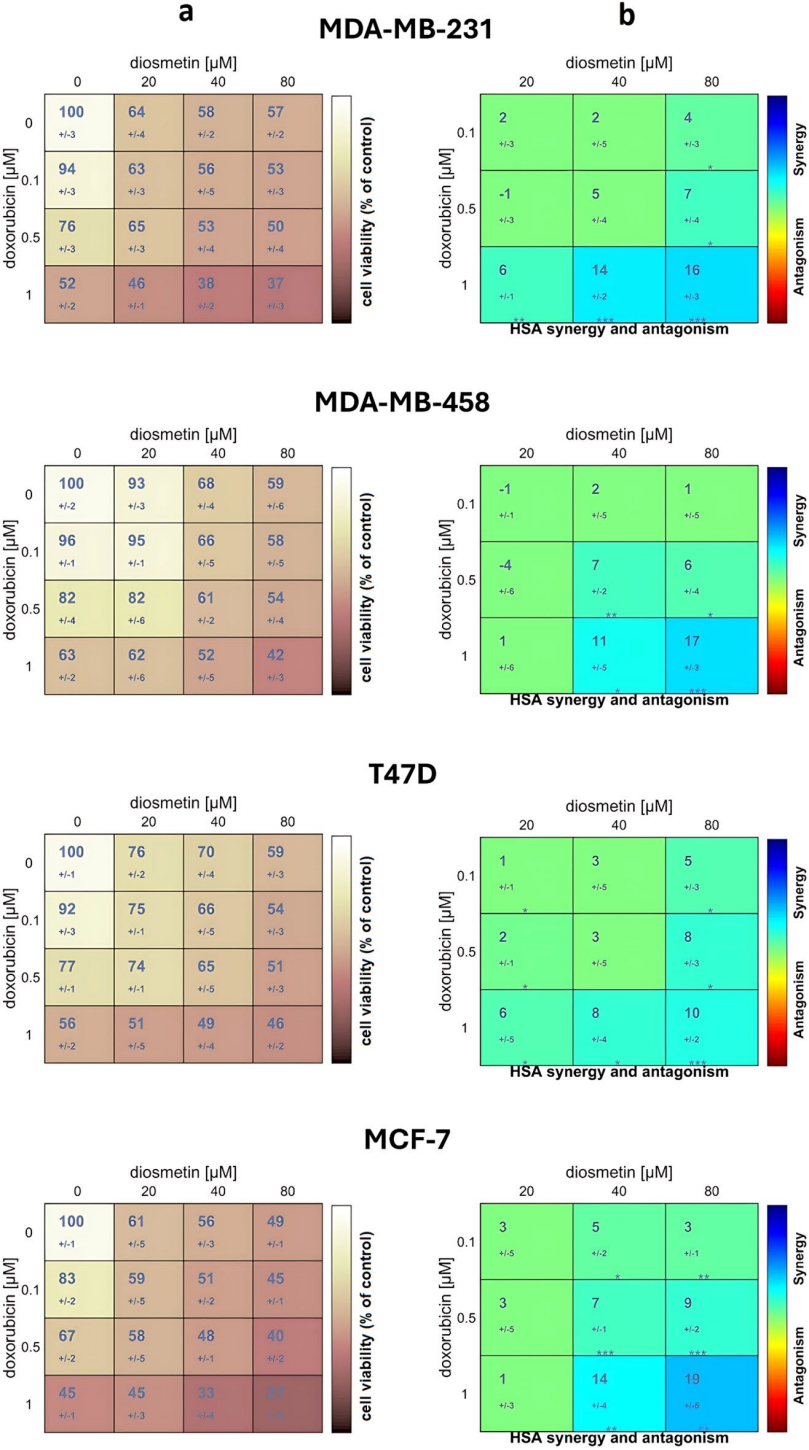
(**a**) Cytotoxicity of DOX (0.1–1.0 µM), DT (20–80 µM) or combined, based on MTT test results after 48 h of incubation. Control cultures were treated with DMSO (0.5%) as a vehicle. The data derived from three independent experiments were reported as the mean ± SD. (**b**) Heat maps of drug-drug interactions analysed using the Highest Single Agent (HSA) model, numerical synergy scores (mean ± SD) are shown within each cell of the heatmaps (negative scores indicate antagonism, positive values indicate synergy), (*//*) z-score level that indicates a significance of the deviation of the observed effect from the expected value according to the HSA model.

The results of the MTT assay showed that DT reduced cell viability in all cell lines tested, but only in the case of MCF7 an IC_50_ value (69.2 µM) was reached (Fig. 1a). Synergy analysis of DOX and DT was performed using Combenefit. The percent inhibition values were used to construct an effect matrix which was analysed using the Highest Single Agent (HSA) model (Fig. 1B). This model assumes that the effect of the combination should not exceed that of the more potent drug in the combination. Heat maps of drug-drug interactions and quantitative synergy indices were obtained to assess the nature and strength of the interaction of the compounds studied.

The results of the synergy analysis of DOX and DT using the HSA model showed a clear synergistic interaction at several dose combination points for all lines tested. The highest synergy values were observed for combinations of medium and high concentrations of DOX (0.5 and 1.0 µM) with higher concentrations of DT (40 and 80 µM). The highest synergy score was shown for the MCF7 cell line (19 vs. 17 for MDA-MB-458, 16 for MDA-MB-231 and 10 for T47D, Fig. 1b). Based on the results of the MTT assays, 80 µM DT, 1 µM DOX, and their combination demonstrated the strongest cytotoxic effects on the MCF-7 cell line and were thus selected for further studies. DOX at 1 µM was selected based on its submaximal cytotoxic effect (IC50 = 0.92µM) ensuring that the observed enhancement by DT reflects true potentiation, not merely additive cytotoxicity. Accordingly, the MCF-7 cell line was chosen for an in-depth analysis of the mechanisms of action of these compounds.

### Apoptosis detection

**Fig. 2:**
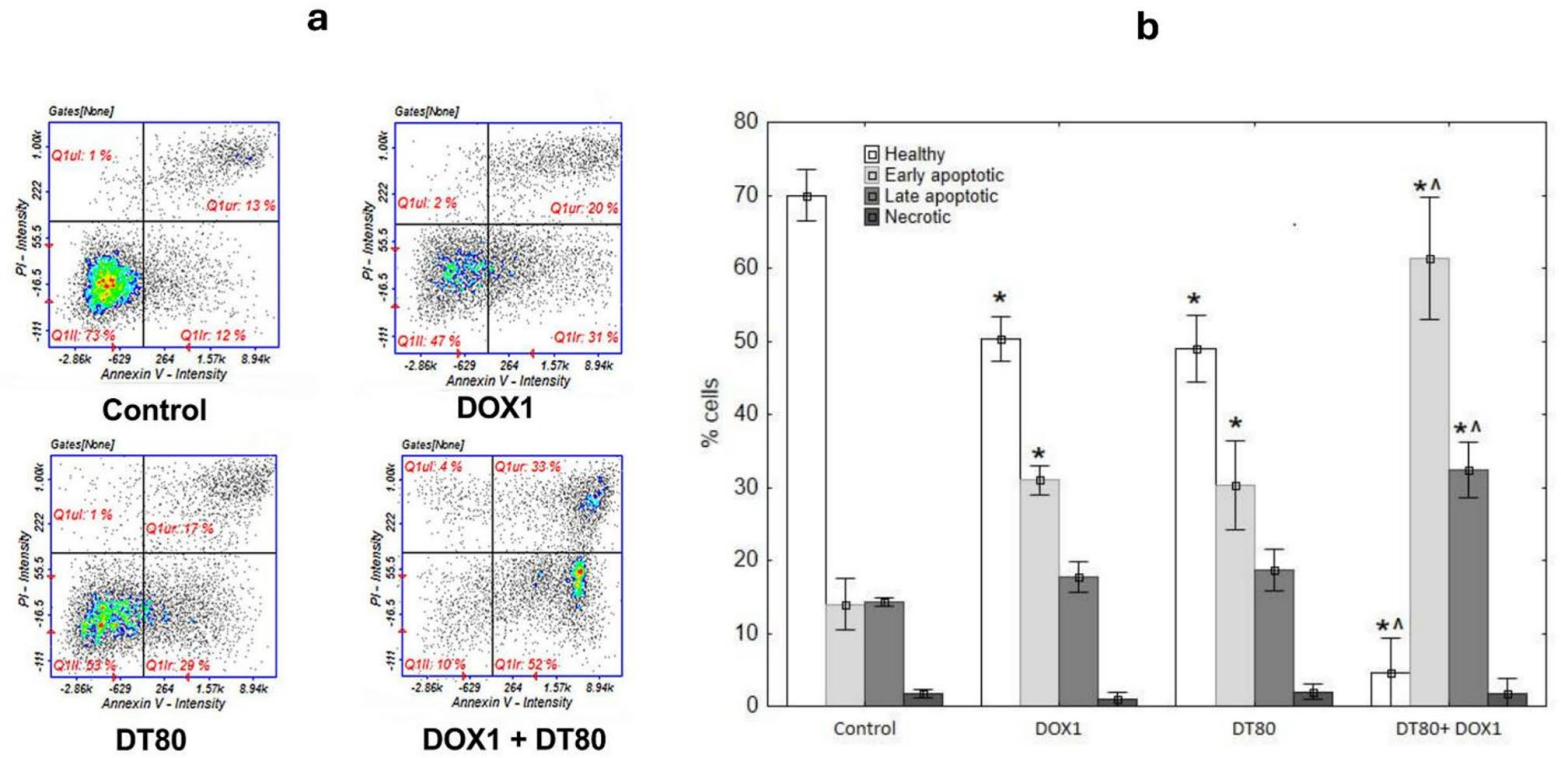
Apoptosis/necrosis detection by image cytometry. MCF-7 cells were treated with 1 µM DOX or 80 µM DT and combined, after 48 h incubation. Control cultures were treated with DMSO (0.5%) as a vehicle **a**. Representative histograms of three independently performed. Q1II—live, Q1Ir—early apoptotic, Q1ur—late apoptotic, and Q1uI—necrotic cells. **b**. The data derived from three independent experiments reported as the mean ± SD. DOX: doxorubicin; DT: diosmetin; DT + DOX: diosmetin + doxorubicin. *p* < 0.05 vs. control, ^*p* < 0.05 vs. DOX alone.

Detection of apoptosis by imaging cytometry showed that after 48-hour incubation with DT, populations of early and late-apoptotic cells were present (30.33 ± 6.11and 18.67 ± 2.88%, respectively). A similar distribution of apoptotic cells was observed in cultures treated with DOX(31.00 ± 2.00% early and 17.67 ± 2.08% late apoptotic cells). The combination of tested compounds significantly increased both populations of apoptotic cells – early apoptotic to 61.33 ± 8.32 and late apoptotic cells to 32.34 ± 3.78%. Necrotic fractions were consistently low in all cultures (Annexin⁻/PI⁺): control 1.67 ± 0.52%, DOX 1.0 ± 0.89%, DT 2.0 ± 0.89%, DT + DOX 1.67 ± 1.86%, suggesting that the cytotoxicity observed is primarily apoptotic cannot be attributed to non-specific membrane damage (Fig. 2).

### Comet assay

The neutral comet assay is a reliable and accurate sensitive tool for the identification and quantitation of double-strand DNA breaks (DSBs) at the single-cell level. The neutral comet assay specifically detects DSBs because it preserves the integrity of double-stranded DNA during lysis and electrophoresis. Unlike alkaline comet assays, which can also detect single-strand breaks (SSBs) but may not differentiate them from DSBs, the neutral conditions prevent the denaturation of DNA, allowing for a clearer assessment of double-strand integrity^26^.

**Fig. 3:**
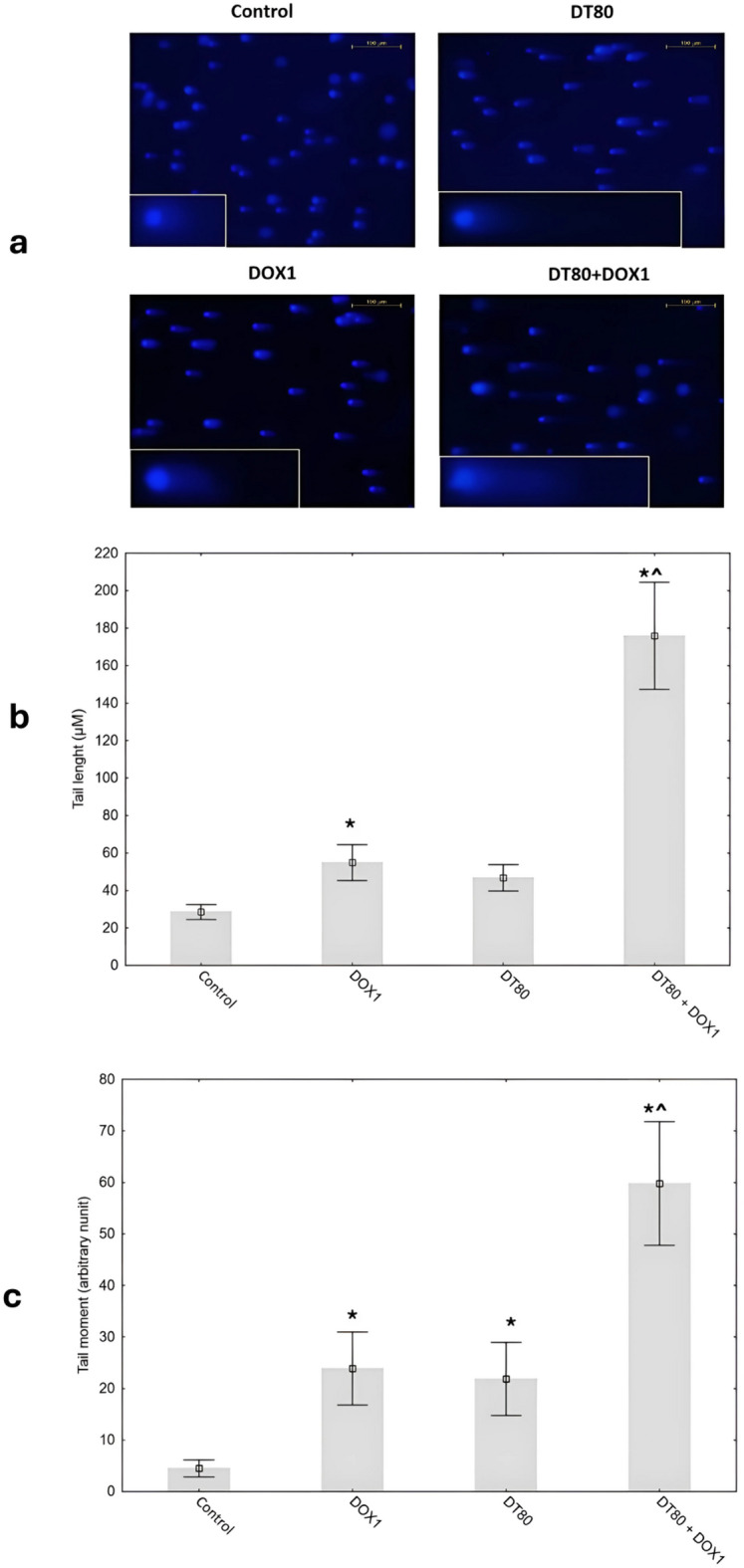
DNA damage induced by DT, DOX and both simultaneously detected by the neutral comet assay in MCF-7 cells. (**a**) Representative micrographs of comet assay results. MCF-7 cells were treated with 1 µM DOX or 80 µM DT and combined for 48 h, control cultures were treated with DMSO (0.5%) as a vehicle (acquired at 100x and 200x magnification). (**b**) Representative graph obtained by plotting the means of tail length and tail moment (**c**). The values are presented as the mean of three independent experiments ± SD. 100 individual cells were selected for calculations. *DOX* doxorubicin, *DT* diosmetin,*DT + DOX* diosmetin + doxorubicin.

DOX treatment as well as DT alone led to DNA damage in MCF-7 cells, as evidenced by the significant increase in the comet parameter (tail length, tail moment) as compared to the control group. Co-administration of DT along with DOX, significantly elevated DOX-induced DNA damage (Fig. 3). To confirm consistency between the two sets of readings, we assessed the correlation between tail length and tail moment in all samples. A strong positive correlation was observed (Pearson’s *r* = 0.89, *p* < 0.001), which confirms that both parameters accurately reflect DSB accumulation in this study.

### Molecular docking results

DT’s bioactive conformations and their selectivity for the P-gp transporter, which are viable targets for reducing MDR in DOX therapy, were evaluated by employing molecular docking to determine the docking scores of the interaction between DT and P-gp.

**Fig. 4:**
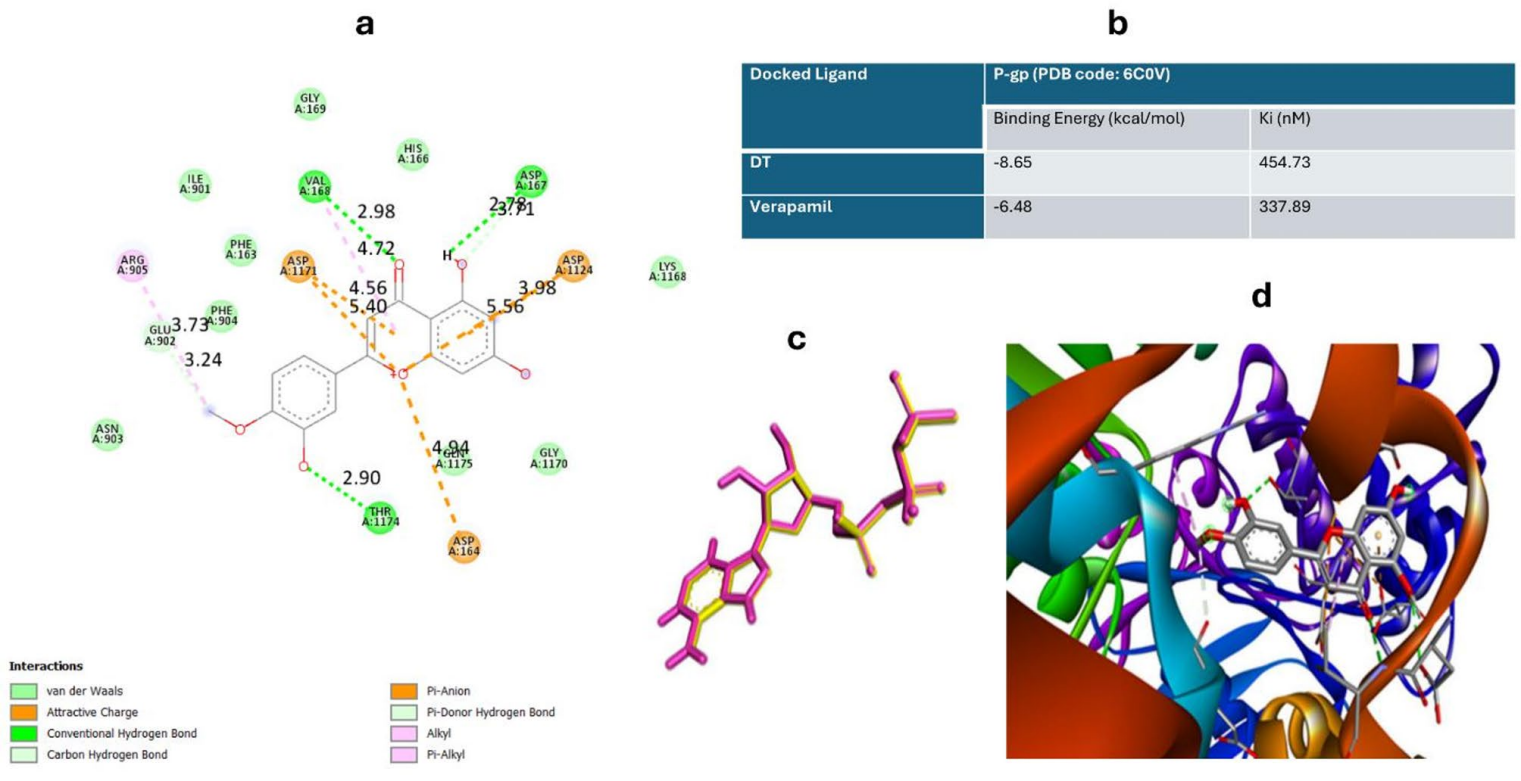
(**a**) 2D scheme of the P-gp complex with DT, (**b**) Binding energy values obtained during docking analysis of DT with P-gp. Verapamil, standard reference P-gp inhibitor was selected as the control ligands, (**c**) Overlying of ligands. Overlying of real (yellow-colored) and predicted position (pink-colored) of the ligand DT (RMSD = 0.936) P-gp (PDB code: 6C0V), (**d**) 3D representation of diosmetin (DT) docked in the binding cavity of P-glycoprotein (P-gp, PDB: 6C0V). Hydrogen bonds (dashed yellow lines) and hydrophobic interactions with key residues are indicated. Visualization performed using Discovery Studio Visualizer^23^.

The results of the docking study are displayed in terms of binding energies and Estimated Inhibition Constant, Ki. out of 30 docking modes analyzed by LGA cluster analysis, the mode with the lowest energy and its respective Ki prediction was chosen for each docking DT and exhibited favorable in silico simulations results with a value of binding energy − 8.65 kcal/mol and inhibition constant 454.73 nM (Fig. 4b). RMSD of re-docked native ligand = 0.936 Å, confirming docking accuracy (Fig. 4c). Verapamil, a well-known P-gp inhibitor, was selected as the control ligands. It was observed that DT exhibited appropriate affinity (−8,65 kcal/mol) to drug binding pocket of P-gp when compared with verapamil that showed binding affinities of −6.48 kcal/mol (Figure Xb). Molecular docking simulations using AutoDock 4.2 revealed that DT exhibited strong binding affinity to P-gp (binding energy: −8.65 kcal/mol), forming hydrogen bonds with VAL168, ASP167, and THR1174 (Fig. 4a). These interactions suggest stable complex formation and support DT’s role as a potential P-gp inhibitor. The 3D visual binding model of DT in the active cavity of P-gp is shown in Fig. 4d.

Molecular docking was implemented for DT to gain a thorough insight into the prospective paths of action to improve DOX chemotherapy efficacy by strongly suppressing the P-gp transporter Hence, it may be assumed, that the properties of DT associated with enhanced DOX cytotoxicity are probably related to the activity of P-gp proteins.

The results from the in silico docking simulations suggest that DT is a promising base for the exploration of new molecules targeting P-gp as potent anticancer agents. These results provide a research basis for our hypothesis, and we performed in vitro experiments to validate specific mechanisms of DT.

### Hoechst accumulation assay

**Fig. 5:**
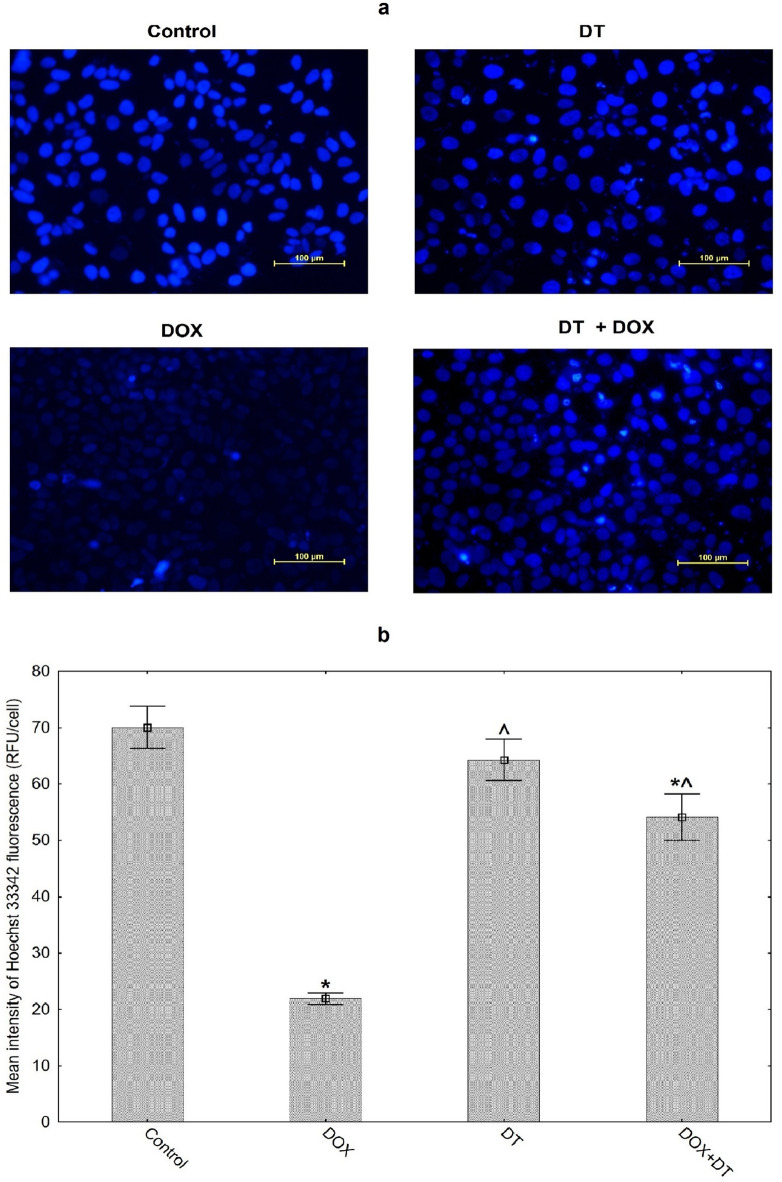
Evaluation of P-glycoprotein (P-gp) activity in MCF-7 cells using Hoechst Accumulation Assay. (**a**) Representative micrographs of cells stained with Hoechst33342. MCF-7 cells were treated with 1 µM DOX or 80 µM DT and combined for 48 h. Control cultures were treated with DMSO (0.5%) as a vehicle (acquired at 200x magnification) (**b**) The intensity of fluorescence, measured by Nikon Eclipse Ti inverted fluorescence microscope. For each sample, three random fields of view were selected, and in each field, 20 nuclei were analyzed. The values are presented as the mean of three independent experiments ± SD. DOX: doxorubicin; DT: diosmetin; DT + DOX: diosmetin + doxorubicin. *p* < 0.05 vs. control, ^*p* < 0.05 vs. DOX alone.

In the Hoechst Accumulation Assay, the weakest fluorescence signal was observed in the group of cells treated with DOX (21.90 ± 3.65 vs. 70.04 ± 13.17 RFU/cell in control cells, Fig. 5b), which may indicate high P-gp activity in MCF-7 line cells treated with the chemotherapeutic agent. However, DT administration led to a notable rise in intracellular fluorescence compared to very faint signals derived from DOX treated group (64.28 ± 13.19 RFU/cell). The obtained images were similar to those that were obtained from the control group (Fig. 5). A strong fluorescence signal after simultaneous administration of DT and DOX suggests that the tested flavonoid inhibits the enhanced P-gp activity caused by the administration of DOX.

### Expression *of ABCB1*/P-gp at the mRNA and protein levels

**Fig. 6:**
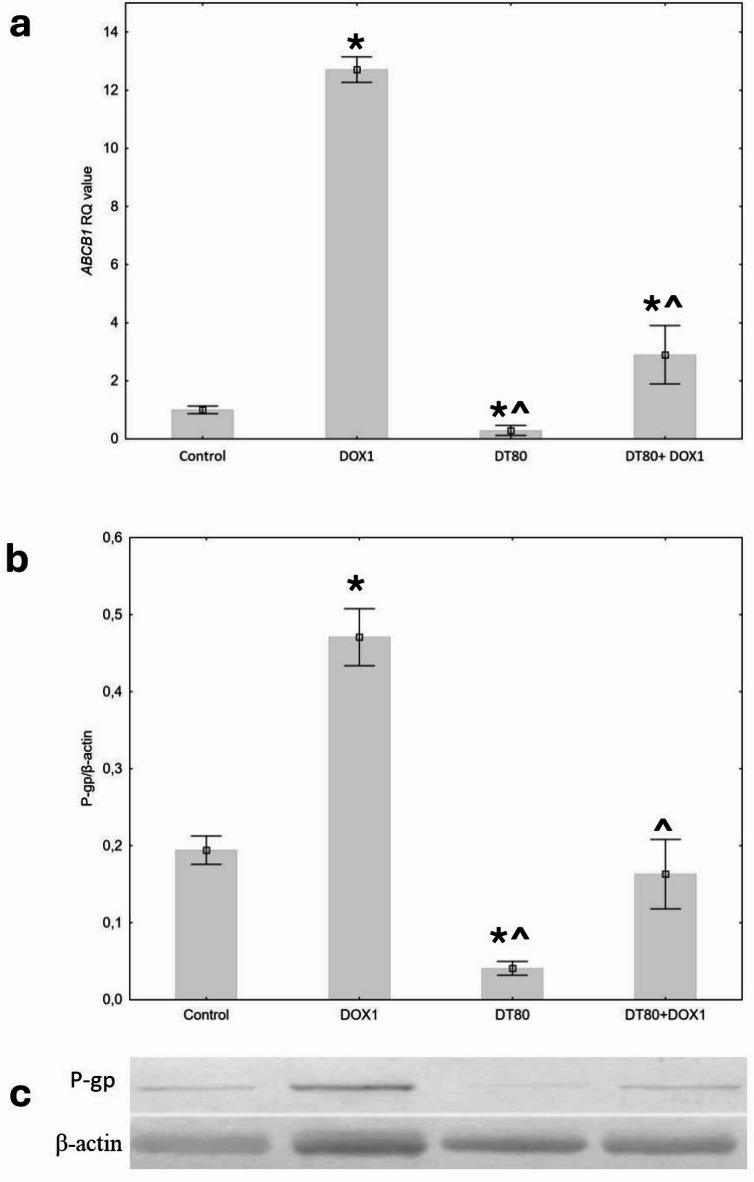
(**a**) Relative mRNA expression level of *ABCB1*. The results were calculated as RQ values and presented as the mean ± SD values of three independent experiments. *ACTB* and *18SN5* were used as reference genes. (**b**) Relative expression level of P-gp protein normalized to β-actin. (**c**) Representative western blot (cropped for clarity). Original blots are presented in Supplementary Fig. 1S. Control cultures were treated with DMSO (0.5%) as a vehicle The cells were treated for 48 h with 1 µM of doxorubicin (DOX) and 80 µM of diosmetin (DT), or combined (DT + DOX). *p* < 0.05 vs. control, ^*p* < 0.05 vs. DOX alone.

mRNA expression analysis showed a more than 12-fold increase in mRNA expression for *ABCB1* after incubation of MCF-7 cells with DOX. In contrast, DT decreased the expression of this gene to a level of approximately 25% of control. Co-incubation of cells with DOX and DT resulted in a more than 4-fold decrease in expression compared to the effect of DOX alone (Fig. 6a). Analysis of P-gp expression (normalized to B-akt) revealed low levels of this protein in control cells, with a significant increase observed after incubation with DOX. As with mRNA levels, DT reduced the level of the protein under study, both when used alone and in combination with DOX (Fig. 6b). The data obtained indicate that the regulation of P-gp expression under experimental conditions occurs primarily at the transcription level.

## Discussion

DOX, as an anthracycline antibiotic, has been widely recognized for its efficacy in breast cancer treatment due to its mechanisms of action, which include DNA intercalation and the generation of free radicals^27,28^. Despite its effectiveness, the clinical application of DOX is limited by its side effects, such as cardiotoxicity, and the development of drug resistance, which significantly diminishes its therapeutic potential^29,30^. Therefore, enhancing the sensitivity of breast cancer cells to DOX may lead to more effective treatment regimens.

Among the natural compounds with beneficial effects on human health, DT has attracted considerable interest because of its potent antioxidant^31,32^ and anti-inflammatory^33^ properties, as well as its ability to inhibit cancer growth. The described properties of these substances suggest that the use of preparations that improve circulation may have a role in the prevention of various types of cancer. Previous studies have revealed that DT exhibits antitumor properties through the suppression of cell proliferation, activation of cell apoptosis, and regulation of the cell cycle in various cancer cell types, including breast cancer, liver cancer, colon cancer, leukemia, and other tumor cells^3–9^ Unlike other flavonoids primarily studied in vitro, DT is already routinely administered in therapeutic settings in chronic venous insufficiency where it affects approximately 30–40% of adults^10^. Undoubtedly, this state of affairs, relatively low price and intensive advertising has an impact on the popularity of preparations containing diosmin. Chronic venous disease often co-exists with breast cancer. The presence of breast cancer was linked to simultaneous chronic venous clinical symptoms in patients with positive estrogen receptor status^14^.Therefore, it is presumed that patients with this oncological disease can often support their treatment with readily available diosmin preparations. So far, it has not been demonstrated what effect preparations commonly used in chronic venous insufficiency have on the development of breast cancer. Based on these findings, DT has emerged as a promising therapeutic candidate for cancer treatment.

The study investigated the effects of DT and DOX on breast cancer cells, demonstrating that this flavonoid substantially enhances the anticancer activity of DOX. Notably, treatment with 80 µM DT and 1 µM DOX significantly reduced MCF-7 cell viability, indicating increased cytotoxicity compared to DOX alone. Although the in vitro concentration of 80 µM used in our study is considerably higher than the concentrations of free diosmetin typically found in human plasma following diosmin intake^34^ this discrepancy can be explained by diosmetin’s metabolism in vivo. Like most flavonoids, diosmetin undergoes extensive first-pass metabolism, resulting mainly in glucuronide conjugates, such as diosmetin-3-O-glucuronide, that circulate in plasma^35,36^. Consequently, the concentration of free (aglycone) diosmetin in serum is generally low. However, increased β-glucuronidase activity in the tumour microenvironment can locally cleave these conjugates, regenerating the active aglycone form and thereby elevating diosmetin concentrations specifically within tumour tissue^37^. This tumour-selective enzymatic hydrolysis process supports the translational relevance of our findings, suggesting that pharmacologically effective diosmetin concentrations can be achieved locally despite low systemic aglycone levels.

DT increases the percentage of apoptotic cells compared to cells treated with DOX alone Our results also showed that DT induces DNA double-strand breaks and significantly increases DOX-induced DNA damage, as confirmed by comet assay. Molecular docking studies suggested that DT may interact favorably with P-gp, potentially offering a strategy to address multidrug resistance. These findings underscore the potential of DT as a sensitizing agent in DOX-based chemotherapy regimens.

These results suggest that DT holds potential as an adjunctive agent requiring further preclinical and in vivo validation. The current study sought to determine DT’s impact alone and in combination with DOX on the proliferation of human breast cancer cells. The cytotoxicity assay proved that DT decreased the growth of all examined breast cancer cell lines, particularly the most significant effect was observed in MCF-7 cells (Fig. 1a). The proven high anti-proliferative capabilities are consistent with previous research that has shown that the growth of breast cancer is substantially suppressed by DT^3,38^. Additionally, this study explored the effect of DT on DOX cytotoxicity in breast cancer cells. Based on the MTT assay, we found that DT significantly increased the cytotoxicity of DOX in all analyzed breast cancer cell lines. The strongest effect was also observed in MCF-7 cells (Fig. 1b).

Moreover, DT has demonstrated promising anticancer properties with a favorable safety profile. Multiple studies have shown that DT selectively inhibits the proliferation of various cancer cell lines, while exerting minimal cytotoxic effects on corresponding normal cells such as mammary epithelial cells, lung epithelial cells, and colon epithelial cells. These findings underscore the potential of DT as a selective anticancer agent that minimizes damage to healthy tissue, thereby positioning it as a promising candidate for further investigation in cancer therapeutics^5,38,39^.

Previous research has demonstrated that DT effectively collaborates with anticancer medications in different cancer cell types. According to research, the simultaneous use of 5-FU and DT has a beneficial effect on inhibiting the proliferation of HCT116 cancer cells. This co-administration has the potential to mitigate the negative side effects of 5-FU while increasing its effectiveness against cancer by triggering apoptosis^40^. Moreover, it has been shown that through the reduced activity of Nrf2 and inhibition of the PI3K/Akt/GSK-3 pathway, the simultaneous administration of DT and paclitaxel effectively caused cell death in non-small cell lung cancer cells^41^.

The comet assay results confirmed the presence of DNA damage after DT treatment. This is consistent with previous studies where DT caused DNA damage in cells of various cancers^42,43^. They indicated that DT not only induced DNA damage itself but also significantly enhanced the damage caused by DOX treatment, resulting in the longest comet tails in MCF-7 cells (Fig. 3). These findings confirm that DT can exacerbate DNA damage in cancer cells while amplifying the effects of DOX. It may be inferred that the accumulation of DNA strand breaks ultimately leads to apoptosis, and therefore, the increased cytotoxicity observed with the combined treatment of DOX and DT likely originates from this enhanced generation of DNA damage.

Interestingly, *in sillico* study revealed a possible interaction between DT and P-gp. P-glycoprotein (P-gp), encoded by the ABCB1 gene, is a well-known efflux pump that significantly contributes to MDR. Several studies have highlighted the significance of P-gp in mediating chemoresistance in various types of cancers. Specifically, increased expression has been noted in numerous malignancies, including breast cancer^44^. The protective function of ABCB1 against the cytotoxic effects of chemotherapeutic drugs has been demonstrated in several cell lines, including MCF-7^45^. The mechanisms underlying P-gp-mediated chemoresistance involve the active efflux of anticancer agents from malignant cells, resulting in reduced intracellular levels of medications and diminished drug efficacy^46^. Upregulation of ABC transporters can result in resistance to traditional chemotherapeutics, including DOX^47^.

It has been shown that DOX can activate glycoprotein-p in cancer cells^48^. In our study, a significant increase in mRNA and protein expression as well as glycoprotein-p activity was observed after incubation of breast cancer cells with DOX. Thus, inhibiting drug transport or manipulating its expression in cancer cells represents a potent strategy to counteract MDR in cancer. Flavonoids have been identified as potent P-gp inhibitors capable of reversing MDR in cancer cells by binding to transporters and disrupting drug efflux^24,49^. Further research has demonstrated that DT and similar compounds can counteract MDR, thereby enhancing the efficacy of chemotherapeutic agents^40,41,50^. However, specific pathways of how DT affects ABC transporters remain unclear.

To confirm our initial in silico study, we performed a Hoechst 33,342 accumulation assay to assess P-gp activity in the examined cells. According to previous study High P-gp activity leads to lower levels of H342 accumulation. Consequently, H342 can be effective in identifying cells resistant to multiple drugs because it possesses qualities typical of P-gp substrates and is particularly suited to recognizing elevated resistance levels^51^. Hoechst 33,342 accumulates in the lipid membrane due to its hydrophobicity, but P-glycoprotein eliminates it^52^.

DT blocked the efflux of Hoechst 33,342 mediated by P-gp and increased its accumulation in MCF-7 cells, as observed under a fluorescence microscope. (Fig. 5). Our results are also consistent with the study of the properties of another flavonoid quercetin, which also decreased Hoechst 33,342 efflux by suppressing P-gp activity^53^.

Furthermore, we have shown that DT affects not only P-gp activity but also expression at the mRNA and protein level, significantly decreasing both baseline in MCF-7 cells and those significantly increased by DOX (Fig. 6).

Although our study demonstrates that DT significantly reduces ABCB1/P-gp expression and functionally inhibits P-gp-mediated efflux—as shown by increased Hoechst 33,342 accumulation—definitive genetic evidence linking P-gp inhibition to enhanced DOX efficacy would provide further clarity. We recognize that approaches such as ABCB1 gene knockdown or knockout are valuable strategies to directly assess the causal role of P-gp in mediating resistance to DOX and its modulation by DT. These experiments would allow to determine whether DT still potentiates DOX cytotoxicity in the absence of functional P-gp and to what extent other pathways might be involved. While these studies were beyond the scope of the current investigation, they represent a critical next step.

While our results demonstrate that DT significantly downregulates ABCB1 mRNA expression, the exact mechanism remains to be elucidated. Flavonoids have been shown to inhibit transcription factors such as NF-κB and AP-1, which regulate ABCB1 promoter activity^54,55^. Additionally, flavonoids may suppress ABCB1 through modulation of the PI3K/Akt/Nrf2 pathway, a known regulator of multidrug resistance genes^56,57^.

Beyond ABCB1, other members of the ATP-binding cassette family, such as ABCG2 (BCRP) and ABCC1 (MRP1), play pivotal roles in multidrug resistance, particularly in breast cancers^58^. Flavonoids have demonstrated modulatory effects on these transporters^59,60^. Although not assessed in the present study, it is plausible that DT may also interact with BCRP or MRP1, potentially amplifying its capacity to reverse MDR.

This research aimed to examine the anti-cancer potential of DT and to explore its underlying mechanisms in breast cancer cells, specifically focusing on its augmented effects with DOX. Previous studies confirmed DT’s capacity to trigger apoptosis and act as an anticancer agent in various cancer types. However, its impact on breast tumors and the development of MDR remains unclear. According to our research, DT shows potent therapeutic activities on breast cancer cell lines, with a particularly strong effect observed in the MCF-7 cell line. Significantly, we showed that DT treatment enhanced the anticancer activity of DOX. This study revealed that DT improved the efficacy of DOX by strongly suppressing *ABCB1* mRNA/protein levels and P-gp activity, thereby outlining a new approach to overcoming MDR in cancer cells. However, the augmentation of DOX cytotoxicity by DT is not solely due to the inhibition of the ABC transporter but also due to the accumulation of DNA damage. This indicates that DT enhances DOX efficacy by promoting DNA damage in tumors. These effects of DT on DOX anticancer properties may consequently augment apoptosis in MCF-7 cells, as confirmed by an appropriate assay. Considering DT’s well-documented safety in chronic venous disease, its repurposing for oncologic indications presents a promising opportunity for accelerated regulatory approval. The use of predictive biomarkers such as elevated ABCB1 expression or baseline P-gp activity may aid in selecting patients most responsive to DT co-treatment.

## Conclusions

To conclude, our findings indicate that DT is a potent candidate for augmenting the effectiveness of breast cancer treatment. Its ability to improve the effects of DOX, overcome MDR, and establish DT as a valuable adjunctive therapy. Our investigations have revealed that DT induces cytotoxicity in breast cancer cell lines, connected with DNA damage. Moreover, it improves the effectiveness of DOX by decreasing the expression and activity of glycoprotein P, thereby increasing the concentration of the chemotherapeutic agent in the cell.

Nevertheless, further in vivo research is essential to fully delineate DT’s impact of DT on breast cancer invasion, hypoxia-related processes, and its role in comprehensive anticancer strategies. While the current study offers strong in vitro evidence that DT enhances DOX efficacy via inhibition of P-gp and induction of DNA damage, we acknowledge that these findings require in vivo validation. Our study not only highlights the potential therapeutic application of DT in improving breast cancer therapies but also advocates for further investigation into clinical applications.

## Supplementary Information

Below is the link to the electronic supplementary material.

Supplementary Material 1

## Data Availability

The datasets supporting the findings of this study can be obtained from the corresponding author upon reasonable request. All relevant data generated or analyzed in this research are fully available within the manuscript [and its supplementary information files].
