# Supplementary material for: Enhancement of doxorubicin efficacy by diosmetin through DNA damage accumulation and P-glycoprotein inhibition in breast cancer cells
Source: Sci Rep. 2025 Aug 21;15:30814. doi: 10.1038/s41598-025-16681-3 (PMC12371033; doi:10.1038/s41598-025-16681-3)
Supplement: Supplementary file 1 — Supplementary Material 1 [file 41598_2025_16681_MOESM1_ESM.docx]

Supplementary Information

Enhancement of Doxorubicin Efficacy by Diosmetin through DNA Damage Accumulation and P-Glycoprotein Inhibition in Breast Cancer Cells

Monika Michalczyk^1^, Ewelina Humeniuk ^1^*, Joanna Kubik ^1^, Grzegorz Adamczuk ^1^, Mariola Michalczuk ^1^, Barbara Madej – Czerwonka ^2^, Maciej Czerwonka^3^ and Agnieszka Korga-Plewko^1^

**a**


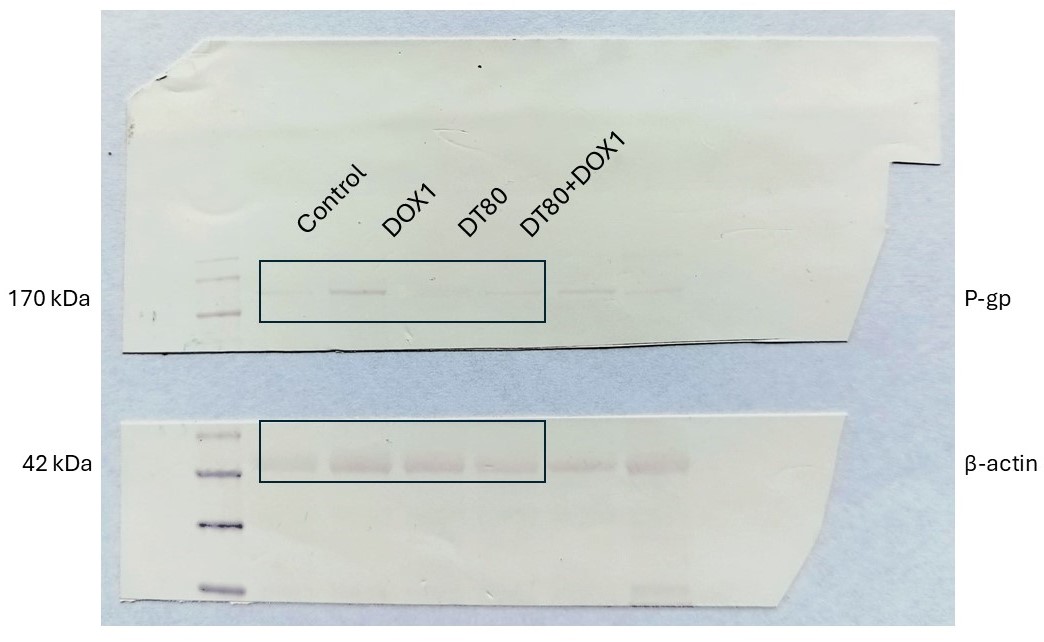


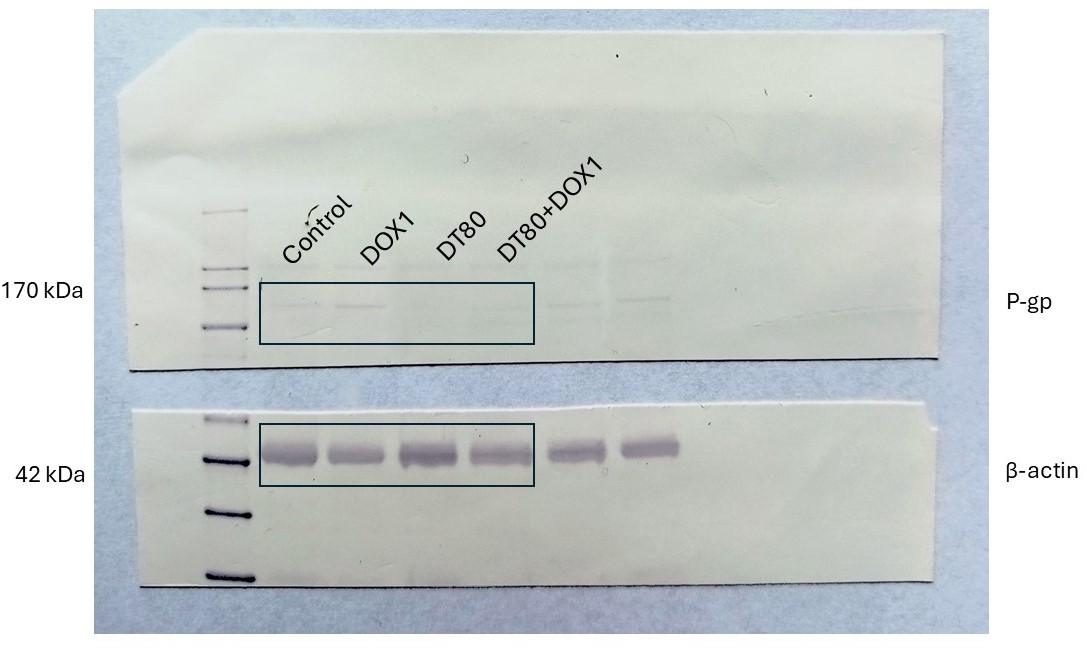


**b**

**c**


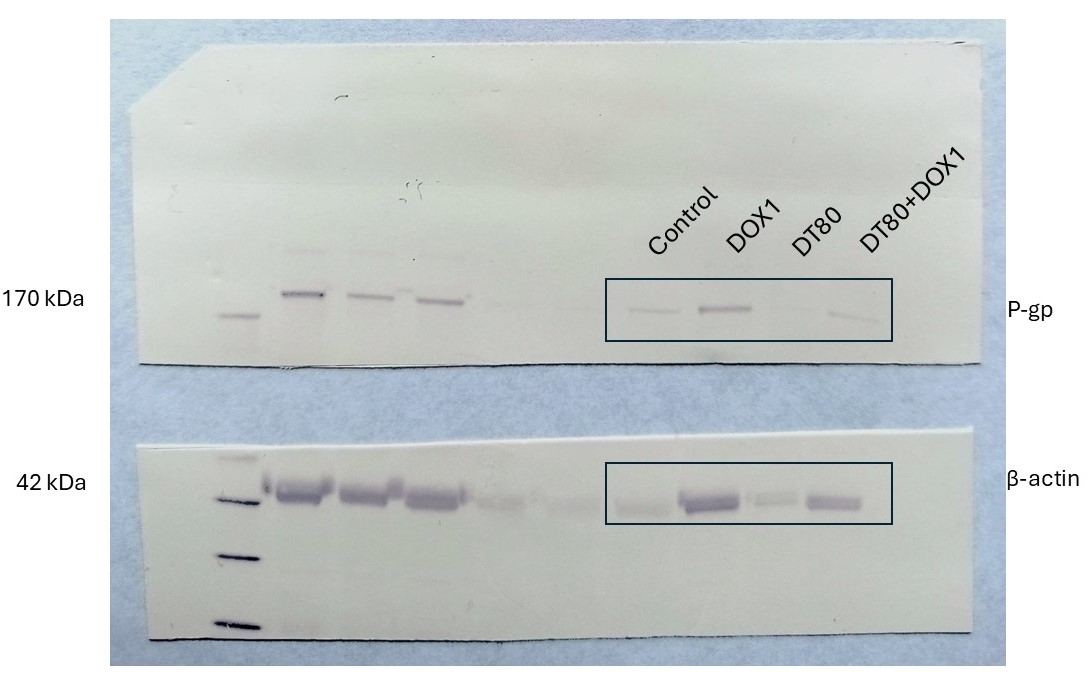


Figure 1S. The original blots used for the evaluation of P-gp protein expression. (**a)** original blots for Fig 1b; **(b, c)** repeated experiments
